# Proteomic Analysis of Intracellular and Membrane-Associated Fractions of Canine (*Canis lupus familiaris*) Epididymal Spermatozoa and Sperm Structure Separation

**DOI:** 10.3390/ani12060772

**Published:** 2022-03-18

**Authors:** Anna Zmudzinska, Mariusz A. Bromke, Rafal Strzezek, Magdalena Zielinska, Beata Olejnik, Marzena Mogielnicka-Brzozowska

**Affiliations:** 1 Department of Animal Biochemistry and Biotechnology, University of Warmia and Mazury in Olsztyn, Oczapowskiego 5, 10-719 Olsztyn, Poland; anna.kuzborska@uwm.edu.pl (A.Z.); rafi@uwm.edu.pl (R.S.); 2 Department of Biochemistry and Immunochemistry, Wroclaw Medical University, Chalubinskiego 10, 50-368 Wroclaw, Poland; mariusz.bromke@umw.edu.pl (M.A.B.); beata.olejnik@umw.edu.pl (B.O.); 3 Department of Systems Engineering, University of Warmia and Mazury in Olsztyn, Heweliusza 14, 10-719 Olsztyn, Poland; m.zielinska@uwm.edu.pl

**Keywords:** epididymal spermatozoa, semen quality, proteomic, sonication, canine

## Abstract

**Simple Summary:**

Epididymal spermatozoa have great potential in current dog reproductive technologies. In the case of azoospermia or when the male dies, the recovery of epididymal spermatozoa opens new possibilities for reproduction. It is of great importance to analyze the quality of the sperm in such cases. Proteomic studies contribute to explaining the role of proteins at various stages of epididymal sperm maturation and offer potential opportunities to use them as markers of sperm quality. The present study showed, for the first time, mass spectrometry and bioinformatic analysis of intracellular and membrane-associated proteins of canine epididymal spermatozoa. Additionally, sonication was used for the separation of dog epididymal sperm morphological elements (heads, tails and acrosomes). The results revealed the presence of differentially abundant proteins in both sperm protein fractions significant for sperm function and fertilizing ability. It was also shown that these proteins participate in important sperm metabolic pathways, which may suggest their potential as sperm quality biomarkers.

**Abstract:**

This study was provided for proteomic analysis of intracellular and membrane-associated fractions of canine (*Canis lupus familiaris*) epididymal spermatozoa and additionally to find optimal sonication parameters for the epididymal sperm morphological structure separation and sperm protein isolation. Sperm samples were collected from 15 dogs. Sperm protein fractions: intracellular (SIPs) and membrane-associated (SMAPs) were isolated. After sonication, sperm morphology was evaluated using Spermac Stain™. The sperm protein fractions were analyzed using gel electrophoresis (SDS-PAGE) and nanoliquid chromatography coupled to quadrupole time-of-flight mass spectrometry (NanoLC-Q-TOF/MS). UniProt database-supported identification resulted in 42 proteins identified in the SIPs and 153 proteins in the SMAPs. Differentially abundant proteins (DAPs) were found in SIPs and SMAPs. Based on a gene ontology analysis, the dominant molecular functions of SIPs were catalytic activity (50%) and binding (28%). Hydrolase activity (33%) and transferase activity (21%) functions were dominant for SMAPs. Bioinformatic analysis of SIPs and SMAPs showed their participation in important metabolic pathways in epididymal sperm, which may suggest their potential as sperm quality biomarkers. The use of sonication 150 W, 10 min, may be recommended for the separation of dog epididymal sperm heads, tails, acrosomes and the protein isolation.

## 1. Introduction

The increasing popularity of dog breeding and the fact that this knowledge may be transferred to endangered Canidae species provides reasons for many scientists to place an increasing emphasis on understanding the specificity of canine reproduction [1]. Epididymal spermatozoa have great potential in current dog reproductive technologies. In the case of azoospermia, or when the male dies, the recovery of the epididymal spermatozoa opens up a new possibility for generating offspring [1,2]. The epididymal spermatozoa may be obtained by various ex vivo or in vivo techniques and then frozen for later use in assisted reproduction technologies [3,4]. Since the dog is also a good model for human reproduction, the acquired knowledge may be used to understand problems in human infertility and improve assisted reproduction methods [5,6].

The sonication is a method used to break cells to isolate their components [7]. It is a physical method based on the phenomenon of cavitation, when the ultrasounds may also affect cell membranes [8]. This method was successfully used to isolate sperm structures, such as the sperm head [9,10], acrosome [11] and tail [12]. Sonication was also used for protein extraction from ejaculated and epididymal spermatozoa of human and stallion subjects [13,14,15]. Sonicated spermatozoa were found to be suitable for the preparation of membrane fractions used to identify proteins that mediate sperm–egg interactions [16].

Proteomic studies contribute to explaining the role of proteins at various stages of epididymal sperm maturation, capacitation, acrosomal reaction and sperm–egg fusion [17,18]. Bioinformatic analyses based on gene ontology provide insight into the protein localization, distribution and participation in exact metabolic pathways [19]. Knowledge of the functions of proteins offers potential opportunities to use them as markers of biological value in reproductive processes or as future components of contraceptives [18,20]. The search for marker proteins associated with the process of sperm preservation is of great importance as it allows the identification of males or semen samples with greater or lesser suitability in this regard [21,22]. Seminal plasma and ejaculated sperm proteome was proposed by Aquino-Cortez et al. [23] and Araujo et al. [24,25]. However, according to the authors’ knowledge, the proteomic study of dog epididymal spermatozoa was not shown until now. Epididymal spermatozoa are different from those ejaculated because they are covered only by epididymal fluid proteins and do not come into contact with accessory glands-secreted proteins. To understand the epididymal sperm fertilizing potential and the role of particular proteins in it, it would be necessary to provide proteome analysis together with recognition of most important sperm metabolic pathways responsible for the quality of the sperm.

Since there are no published reports in this field, it was hypothesized that intracellular and membrane-associated fractions of canine epididymal spermatozoa were differentially composed and that the application of optimal sonication parameters could be established for the separation of the morphological structures of epididymal spermatozoa and protein isolation.

Therefore, the aim of this study was to (1) provide the proteomic analysis of intracellular and membrane-associated fractions of canine (*Canis lupus familiaris*) epididymal spermatozoa and (2) to find optimal sonication parameters for the epididymal sperm morphological structure separation and sperm protein isolation.

## 2. Materials and Methods

### 2.1. Chemicals and Media

All chemicals of the highest purity were purchased from the Sigma Chemical Company (St. Louis, MO, USA) unless otherwise stated.

### 2.2. Animals

The study was performed on 15 mixed-breed dogs (1 to 6 years old; mean 3.5 years) with body weight from 12 to 33 kg (mean 21.5 kg) of unknown fertility. The dogs were fed and kept in the same environmental conditions in the Shelter for Homeless Animals in Tomaryny (Poland). All of the dogs were presented for a routine orchiectomy by a qualified veterinary doctor as a part of the program of preventing animal homelessness and promoting adoption. The consent form was achieved from the director of the shelter.

### 2.3. Cauda Epididymal Semen Collection

The materials, i.e., the testis with epididymis, were placed in sterile plastic containers in 0.9% NaCl solution and then in a thermobox at a temperature of 4 °C and delivered within one hour to the laboratory of the Department of Animal Biochemistry and Biotechnology (University of Warmia and Mazury in Olsztyn, Poland). Immediately after that, the gonads were washed with DPBS (Dulbecco’s Phosphate-Buffered Saline, Gibco, Grand Island, NY, USA). The cauda epididymal tissue was cut carefully with a sterile scalpel to avoid sectioning the blood vessels. The effluent of the epididymal semen was aspirated from the cauda epididymal tissue using an automatic pipette [26] with modifications. Samples obtained from the cauda epididymis (right and left) of the same animal were pooled.

### 2.4. Spermatozoa Quality Assessment

The epididymal sperm concentration was determined using a Bürker chamber under a light microscope (Olympus BX41TF, Tokyo, Japan). The concentration of epididymal spermatozoa in the studied group of dogs ranged from 21.8 to 56.4 × 10^8^ spermatozoa/mL, (36.9 ± 2.3 × 10^8^ spermatozoa/mL, mean ± SE).

The sperm samples were subsequently assessed using a computer-assisted semen analysis (CASA-system, HTM-IVOS, 12.3, Hamilton-Thorne Biosciences, MA, USA). The procedure was described previously by Mogielnicka-Brzozowska et al. [27]. The following software settings recommended by the manufacturer for canine sperm analyses were used: frame acquired—30, frame acquisition rate—60 Hz, minimum cell contrast—75, minimum cell size—6 pixels, straightness threshold—75%, path velocity threshold—100 μm/s, low average path velocity (VAP) cut-off—9.9 μm/s, low straight-line velocity (VSL) cut-off—20 μm/s, static size gates—0.80–4.93, static intensity gates—0.49–1.68, static elongation gates—22–84. The percentages of spermatozoa with total motility (TMOT, %) and progressive motility (PMOT, %), were analyzed in each epididymal sperm sample.

### 2.5. Sample Preparation for the Sonication

For the sonication, the epididymal sperm concentration was established at 1 × 10^8^ spermatozoa in 300 μL DPBS, and samples were stored at 4 °C no longer than two hours until further analyses. The sperm samples were centrifuged at 800× *g* for 10 min, 4 °C to remove epididymal fluid (EF). The remaining supernatant (EF) was removed, and the sperm pellet was resuspended in 1 mL DPBS (Gibco) and again centrifuged at 800× *g* for 10 min, 4 °C, to remove loosely bound proteins [28]. The remaining supernatant was removed and discarded.

The epididymal spermatozoa samples were placed in an ice bath and subjected to sonication using the Omni Sonic Ruptor 250 Ultrasonic Homogenizer (Omni International, Kennesaw, GA, USA). The maximum sonication power was 250 W. The following sonication parameters were used for the experiment variants: C—control (without sonication); S1—5 min, 50 W; S2—10 min, 50 W; S3—30 min, 50 W; S4—10 min, 150 W. The frequency of the ultrasound in all the sonication variants was 60 kHz. Following sonication, the sperm samples were centrifuged (8000× *g* for 10 min at 4 °C). The resulting supernatant containing sperm intracellular proteins (SIPs) was collected into another Eppendorf tube. To the remaining sperm pellet, an aliquot of 1 mL of Radioimmunoprecipitation Assay Buffer (RIPA), containing 50 mM Tris-HCl; 150 mM NaCl; 1% (*v*/*v*) Triton X-100; 0.5% sodium deoxycholate; 0.1% SDS; and ddH_2_O, pH 7.4 was added, and the sample was dissolved, incubated for 5 min at 4 °C, and then vortexed and left overnight in buffer ([27,29]; with modifications). Protease Inhibitor Cocktail (Sigma-Aldrich/P8340, St. Louis, MO, USA) was added both to the SIPs and the remaining sperm pellet. The samples were then centrifuged at 8000× *g* for 10 min, 4 °C to obtain a clear lysate of the sperm membrane-associated proteins (SMAPs). The clear lysate was collected into another Eppendorf tube. The SIPs and SMAPs were frozen and kept at −80 °C until further analyses.

### 2.6. Morphology Assessment of Epididymal Spermatozoa Using Spermac Stain

**Figure 1:**
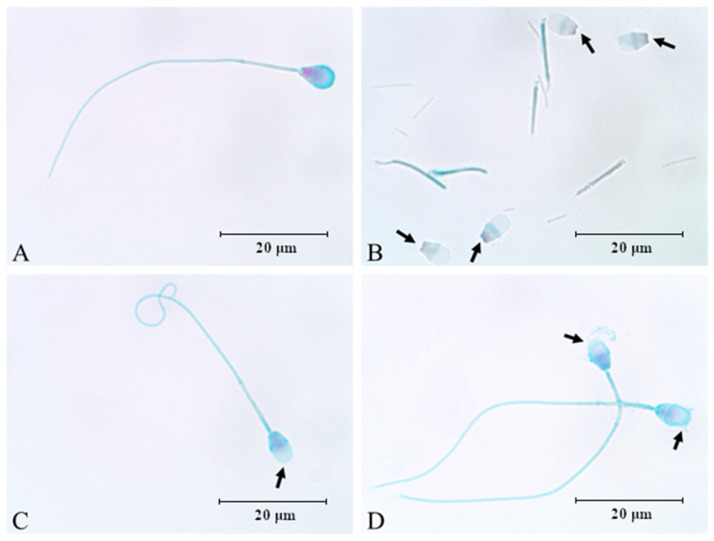
Morphological changes in dog (*Canis lupus familiaris*) cauda epididymal spermatozoa (n = 15) after sonication stained with the Spermac stain (1000× magnification in light microscope). (**A**)—normal epididymal sperm; (**B**)—damaged epididymal sperm, with detached head and detached tail; (**C**)—damaged epididymal sperm showing acrosome loss; (**D**)—epididymal sperm showing damaged acrosomal membrane. Arrows show the exact sperm structures.

The dog epididymal spermatozoa from each variant of the sonication (S1 to S4) were prepared as smears on glass slides using 10 μL of each sample (1 × 10^8^ spermatozoa) and left to dry on a thermoblock (5 min, 37 °C). The control samples were prepared in the same way. Spermac Stain™ (FertiPro, Beernem, Belgium) staining was then performed according to the manufacturer’s recommendations with modifications. The morphological defects caused by sonication in dog epididymal spermatozoa were examined under bright light microscopy at the magnification of 1000× (Olympus BX41TF). Approximately 200 spermatozoa were counted in each sperm sample. The spermatozoa were classified into two categories: undamaged (without secondary defects) or damaged (at least one secondary defect), according to the World Health Organization guidelines [30]. During the determination of the sperm head defects, attention was paid to the shape of the acrosome and continuity of the membranes surrounding the sperm nucleus (Figure 1). The sperm acrosome was found to be normal after dark green staining, and it formed a thin, smooth border at the top of the sperm head (Figure 1A). According to the protocol of Spermac Stain™ (FertiPro), the epididymal sperm fragments were stained as follows: the nucleus (red), the equatorial region (pale green) and the midpiece and the tail (green). When assessing the sperm tail, a detached tail was identified as a defect (Figure 1B). Two types of sperm head defects were recognized: acrosome loss (Figure 1C) and a damaged acrosomal membrane (Figure 1D).

### 2.7. Total Protein Content Measurement

The total protein content was measured using Bradford Reagent (Sigma–Aldrich/B6916) in the control samples (without sonication) and in both the SIP and SMAP fractions subjected to the various sonication variants: S1, S2, S3, S4.

### 2.8. Polyacrylamide Gel Electrophoresis (SDS-PAGE)

For proteomic analysis, the SIPs and SMAPs isolated from the spermatozoa of individual dogs (n = 15) were pooled. Each pool was run in triplicate (technical replicate). The SDS-PAGE procedure was described previously by Mogielnicka-Brzozowska et al. [27]. The molecular weight (MW) and the optical density (OD) of the stained protein bands (PB) were determined using MultiAnalyst 1.1 software (BioRad, Laboratories, Hercules, CA, USA).

### 2.9. Identification of Proteins by Mass Spectrometry

#### 2.9.1. In-Gel Trypsin Digestion

The SDS-PAGE gel samples were processed according to the protocol described by Shevchenko et al. [31]. Briefly, gel pieces with protein fractions (PFs) of SIPs and SMAPs were washed in acetonitrile and 25 mM ammonium bicarbonate to remove Coomassie stain, reduced in 10 mM dithiothreitol and alkylated with 55 mM iodoacetamide. Next, the gel pieces were dehydrated with acetonitrile and dried in a vacuum centrifuge. Subsequently, the gel pieces were rehydrated for 10 min at 4 °C in 10 mM ammonium bicarbonate containing 13 ng/µL trypsin (Promega, Fitchburg, WI, USA). Gel samples were left overnight at 37 °C for complete digestion. After digestion, the peptides were extracted by adding 100 μL of extraction buffer (1:2 [*v*/*v*] 5% formic acid/acetonitrile) to each tube. All of the tubes were vortexed and incubated in a shaker for 15 min at 37 °C. Finally, the peptides were desalted using C18 zip tips (Millipore, Burlington, MA, USA), vacuum-dried and resuspended in water with 0.1% formic acid.

#### 2.9.2. NanoUPLC-Q-TOF/MS Analysis

Waters Acquity liquid chromatography M-Class system (Waters Corp., Milford, MA, USA) equipped with a Peptide BEH C18 analytical column (150 mm × 75 µm; 1.7 µm, Waters Corp., Milford, MA, USA) and Symmetry C18 precolumn (180 µm × 20 mm; 1.7 µm, Waters Corp., Milford, MA, USA) was utilized to separate digested samples.

Each sample was injected on the precolumn and then washed with 99% solvent A (0.1% formic acid in water) at a flow rate of 5 µL/min for 5 min. After washing, the peptides were transferred to an analytical column and separated. The flow rate of the mobile phase was 300 nL/min. The total run time of the analytical gradient, including the column equilibration step, was set at 75 min. The elution gradient steps were as follows: From 0 to 2 min, the concentration of buffer B (0.1% formic acid in acetonitrile) was 5%. Then at 15 min, its concentration was increased to 30%. Next at 45 min, buffer B concentration was increased to 60% and then to 85% at 48 min. During the next 10 min, buffer B concentration was at 85%, before being reduced to 5%, within a time interval of 58 min to 58.5 min.

A mass spectrometry (MS) analysis was performed using Synapt G2-Si (Waters Corp., Milford, MA, USA) with a nanoelectrospray ionization (nESI) source, operating under a positive ion mode. The capillary voltage was set at 3.0 kV, and the cone voltage was set at 40 V. The cone gas flow was set at 40 L/h, and the source temperature was set at 100 °C. The nanoflow gas flow was set at 0.2 Bar. Data were acquired for *m*/*z* 70 to *m*/*z* 1800 using data-independent mode (MSE). Leucine enkephalin (*m*/*z* 556.2771) was used as a Lockspray. The lock mass was acquired every 45 s, and mass correction was applied automatically during acquisition.

Raw chromatography files were analyzed with Byonic software (Protein Metrics, Cupertino, CA, USA). The following settings were used for peak picking and identification: trypsin digestion, max. two miss-cleavages, max. three charges, possible modifications: carbamidomethylated Cys; oxidation of Met and Trp; dioxidation of Trp; pyro-Glu; de-carbamidomethylated Cys; oxidation of Pro; phosphorylation of Ser, Tyr, Thr; (di)methylation of Lys and Arg; acetylation of Lys; trimethylation at Lys; sulfation of Cys, Ser, Thr, Tyr. The detected peptides were compared to SWISSPROT dog proteome (CANLF)-downloaded April 2021. False identifications were limited by comparison with common contaminants and decoys obtained by reverse amino acid sequencing of in silico-cleavage peptide models.

### 2.10. Gene Ontology and Functional Annotation

Functional enrichment of the proteins of the sperm intracellular proteins (SIPs) and sperm membrane-associated proteins (SMAPs) of the canine (*Canis lupus familiaris*) epididymal sperm in Gene Ontology (GO) categories: molecular function, biological process, protein class and pathways were obtained from PANTHER Classification System v. 16.0 (online tools, http://pantherdb.org, accessed on 23 November 2021). A Venn diagram was constructed using web tool (http://bioinformatics.psb.ugent.be/webtools/Venn, accessed on 23 November 2021). GO plots were performed using GraphPad Prism software (GraphPad Prism v. 9.2.0. for Windows, GraphPad Prism software, San Diego, CA, USA).

### 2.11. Statistical Analysis

The data analysis was carried out using Statistica version 13.1 (StatSoft, TIBCO Software Inc., Palo Alto, CA, USA). The results of sonication variants are presented as means with a standard error (mean ± SE). The percentages of sperm morphological defects and protein separation were analyzed with ANOVA (Duncan’s multiple range test). Different sonication variants were compared with their respective control and each by each. A comparison of the OD values of the DAPs was also performed using Student’s *t*-test for independent samples to determine statistically significant differences.

## 3. Results

### 3.1. Sperm Motility Assessment

The sperm cells showed a total motility (TMOT, %) range from 84.0 to 95.0% (90.9 ± 0.9%, mean ± SE), while the progressive motility (PMOT, %) ranged from 41.0 to 72.0% (54.7 ± 9.9%, mean ± SE).

### 3.2. Influence of Sonication on Epididymal Spermatozoa Morphology

The influence of the sonication on the morphological changes in dog epididymal spermatozoa is shown in Figure 1.

**Figure 2:**
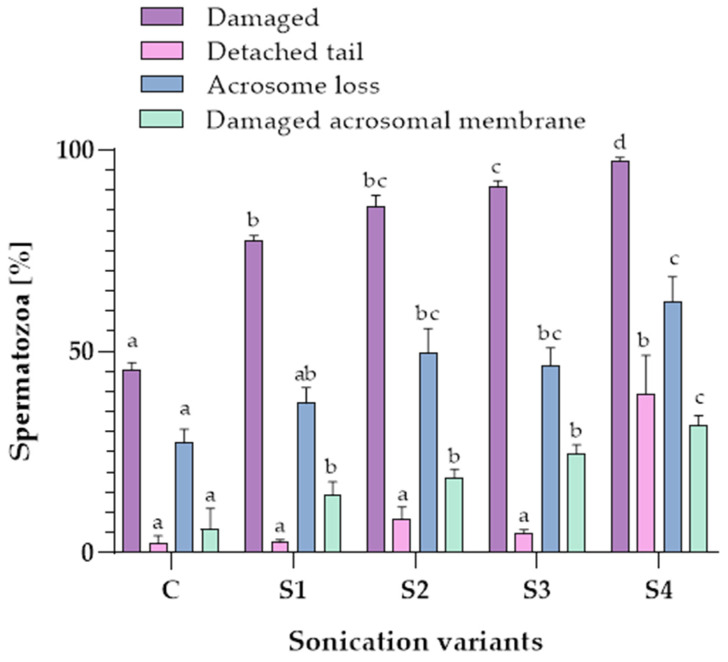
The evaluation of the morphological changes in dog (*Canis lupus familiaris*) cauda epididymal spermatozoa (n = 15) after the treatment with different sonication variants. C—the control sample (without sonication), S1—5 min, 50 W; S2—10 min, 50 W; S3—30 min, 50 W; S4—10 min, 150 W. Values are presented as the mean ± SE. Different sonication variants were compared with their respective control each by each. Different letters indicate significant difference (*p* ≤ 0.05).

Significant differences in the percentage of the total number of damaged spermatozoa were found between the control sample (C) and samples subjected to different sonication variants (S1, S2, S3, S4). After S4 treatment, 97.4 ± 0.8% (mean ± SE) of dog epididymal spermatozoa were found to be damaged. The effect of a sonication power of 150 W applied in variant S4 on the percentage of the total number of damaged spermatozoa was more significant (*p* ≤ 0.05) than sonication power of 50 W in variants S1, S2 and S3 (Figure 2). 

When comparing the occurrence of a detached tail in the spermatozoa samples, it was noted that the highest percentage of the abovementioned defect (39.4 ± 9.6%) (*p* ≤ 0.05) was found after the application of the last sonication variant (S4). The percentage of the detached tail in the S4 variant (with the parameters 10 min, 150 W) was over 25% higher than the other tested variants (C, S1, S2, S3). There were no significant differences between the control and the sonicated samples (S1, S2, S3) (*p* > 0.05) in the percentage of the spermatozoa with a detached tail (Figure 2).

The use of a higher sonication power (150 W) resulted in more spermatozoa losing their acrosomes. The control sample contained 27.3 ± 3.4% spermatozoa without acrosome, but the use of sonication increased the occurrence of this defect to 62.4 ± 6.1% in the S4 variant (*p* ≤ 0.05) (Figure 2).

When comparing the percentage of damaged acrosomal membranes in the spermatozoa samples, it was noted that the highest value of the defect occurred when the last sonication variant (S4) was applied (31.8 ± 2.2%) (*p* ≤ 0.05). The percentages of damaged sperm acrosome membrane in S1, S2, S3 were 14.3 ± 3.3%, 18.5 ± 2.2% and 24.6 ± 2.2% (*p* > 0.05), respectively (Figure 2).

### 3.3. Protein Content in Samples after the Sonication

After sonication, the cauda epididymal spermatozoa proteins were divided into two fractions. The first fraction contained the sperm intracellular proteins (SIPs) that flowed into the solution from the sperm cells that disintegrated during the sonication. The second fraction contained the sperm membrane-associated proteins (SMAPs) obtained after the protein extraction from the epididymal sperm residues remaining after the centrifugation of sonicated samples.

**Figure 3:**
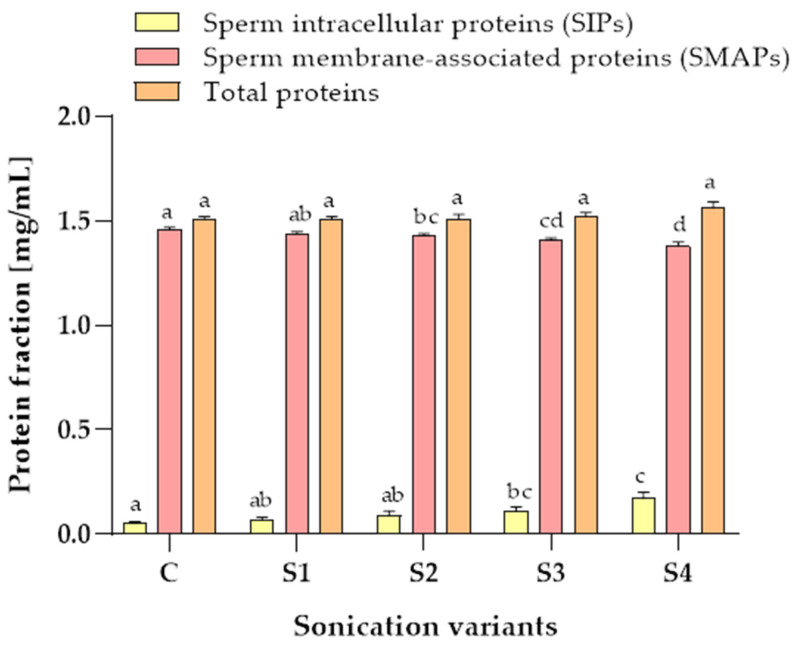
Average amounts of intracellular proteins (SIPs) and membrane-associated proteins (SMAPs) (mg/mL) of dog (*Canis lupus familiaris*) cauda epididymal spermatozoa (n = 15) obtained using different sonication variants. C—control sample (without sonication), the S1—5 min, 50 W; S2—10 min, 50 W; S3—30 min, 50 W; S4—10 min, 150 W. Values are presented as the mean ± SE. Different sonication variants were compared with their respective control each by each. Different letters indicate significant difference (*p* ≤ 0.05).

The protein contents in the SIP and SMAP fractions were interdependent (Figure 3). A higher sonication power (150 W) resulted in more proteins being released from the sperm cells. The control sample contained 0.05 ± 0.01 mg/mL SIPs. The use of sonication slightly increased the content of SIPs in all sonication variants up to 0.17 ± 0.03 in the S4 variant. The content of SMAPs in the control samples was 1.46 ± 0.01 mg/mL. The use of sonication slightly decreased the content of SMAPs in all sonication variants up to 1.38 ± 0.02 mg/mL in the S4 variant (Figure 3).

A comparison of the total protein content (as a sum of the SIPs and the SMAPs) of samples subjected to variants S1, S2, S3, S4 and the control sample indicate that the total protein content was not significantly influenced by sonication power or time (*p* > 0.05) (Figure 3). 

### 3.4. SDS-PAGE Analysis

**Figure 4:**
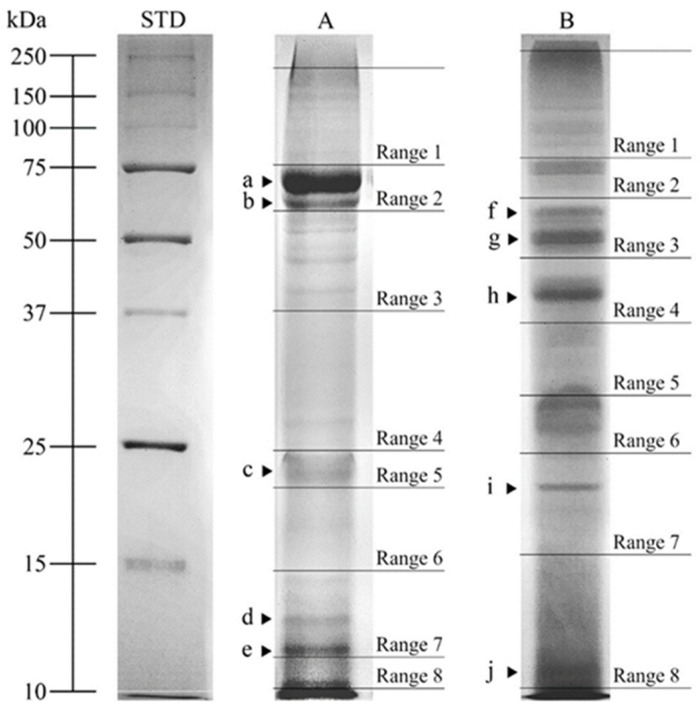
One-dimensional SDS-PAGE (12%). Line A: sperm intracellular proteins (SIPs) of dog (*Canis lupus familiaris*) cauda epididymal spermatozoa (n = 15); Line B: sperm membrane-associated proteins (SMAPs) of the dog cauda epididymal spermatozoa. Proteins were identified using mass spectrometry in ranges 1–8 (Range 1 to 8) for SIPs and SMAPs. Differentially abundant protein fractions were marked with letters from a to j. STD—molecular weight markers.

The SDS-PAGE protein profiles of pooled SIPs and SMAPs were analyzed. The protein profile of the SIPs of the dog cauda epididymal spermatozoa was characterized by the presence of 21 PFs with molecular weights (MW) ranging from 10.6 to 163.2 kDa (Figure 4, Line A). 

The values of MW of PFs on SDS-PAGE gels were averaged for all sonication variants (C, S1, S2, S3, S4). Optical density (OD) analysis in PFs showed higher protein content (*p* ≤ 0.001) for five PFs in the SIPs compared with corresponding PFs of the SMAPs. These were marked with letters from a to e, with the following MW and average optical density (OD) values: (a) 69.0 kDa, 0.36 ± 0.012 OD; (b) 63.6 kDa, 0.32 ± 0.013 OD; (c) 20.2 kDa, 0.29 ± 0.008 OD; (d) 12.8 kDa, 0.28 ± 0.006 OD; (e) 11.9 kDa, 0.30 ± 0.007 OD (Table 1).

The SDS-PAGE profile of the SMAPs of the dog epididymal spermatozoa was characterized by the presence of 19 PFs with MW range from 11.3 to >250.0 kDa (Figure 4, Line B).

The PF molecular weights were averaged for all sonication variants (C, S1, S2, S3, S4). OD analysis in these PFs showed higher protein content (*p* ≤ 0.001) for five PFs in the SMAPs when compared with the corresponding (showing the same MW) PFs of the SIPs. They were marked with letters from f to j, with the following MW and OD values: (f) 61.1 kDa, 0.28 ± 0.006 OD; (g) 50.4 kDa, 0.32 ± 0.008 OD; (h) 40.0 kDa, 0.29 ± 0.014 OD; (i) 18.7 kDa, 0.29 ± 0.006 OD; (j) 11.3 kDa, 0.31 ± 0.013 OD (Table 1).

### 3.5. Mass Spectrometry Analysis

SDS-PAGE protein fractions were analyzed in Ranges 1–8, using NanoUPLC-Q-TOF/MS and UniProt identifiers (Figure 4). Differentially abundant protein fractions are shown in Table 1 and Table 2.

**Figure 5:**
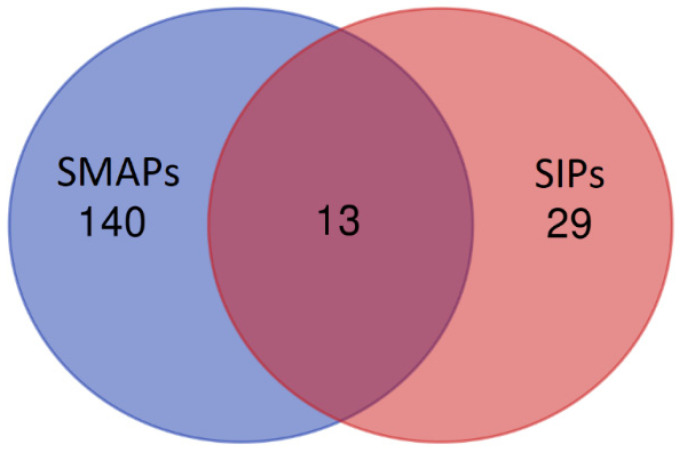
Venn diagram showing the number of proteins identified in the sperm intracellular proteins (SIPs) and the sperm membrane-associated proteins (SMAPs) of the dog (*Canis lupus familiaris*) cauda epididymal spermatozoa.

Ten polypeptides with the highest scores were identified as DAPs in the SIPs fraction: lactotransferrin (*LTF*), carboxylesterase 5A (*CES5A*), albumin (*ALB*), olfactomedin 4 (*OLFM4*), prostaglandin-H2 D-isomerase (*PTGDS*), glutathione peroxidase (*GPX5*), putative lipocalin-like protein (*LCNL1*), putative peptidyl-prolyl cis-trans isomerase (*CSNK1G1*), intracellular cholesterol transporter (*NPC2*) and leucine-rich repeat neuronal protein 3 (*LRRN3*). Eight polypeptides were identified as DAPs in the SMAPs fraction: epididymal secretory protein E1 (*NPC2*), lactotransferrin (*LTF*), cysteine-rich secretory protein 2 (*CRISP2*), WAP domain-containing protein (N/A/WAPdcp), actin, cytoplasmic 1 (*ACTB*), ubiquitin-60S ribosomal protein L40 (*UBA52*), beta-N-acetylhexosaminidase (*HEXB*) and acrosin-binding protein (*ACRBP*). UniProt database-supported identification resulted in 42 proteins identified in the SIPs and 153 proteins in the SMAPs. The proteins (13) present in both SIPs and SMAPs were: *ALB*, *GPI*, *UBA52*, *CRISP2*, *PTGDS*, *LTF*, *GPX5*, *ACRBP*, *LCNL1*, *CES5A*, *OLFM4*, *NPC2*, *HEXB* (Figure 5).

The proteins of the SIPs and SMAPs with significant MS scores are listed in Supplementary Tables S1 and S2, respectively.

### 3.6. Gene Ontology and Functional Annotation

**Figure 6:**
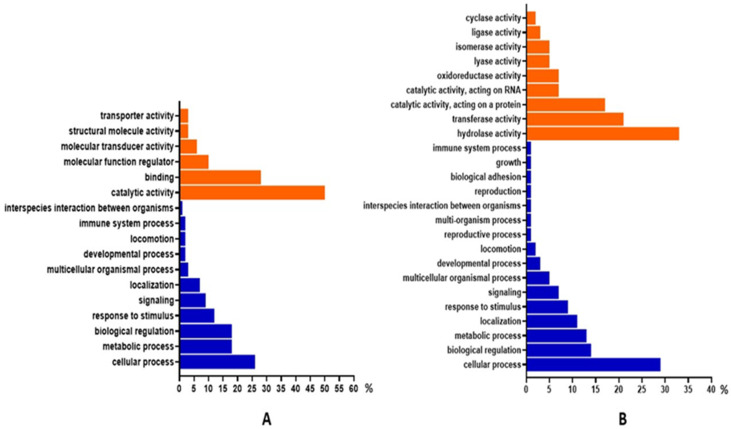
Gene ontology (GO) enrichment of (**A**) sperm intracellular proteins (SIPs) and (**B**) sperm membrane-associated proteins (SMAPs) of the dog (*Canis lupus familiaris*) cauda epididymal spermatozoa. Significant GO terms for molecular function (orange) and biological process (blue) are presented. The analyses were made by the PANTHER Classification System (v. 16).

Based on gene ontology (GO) enrichment, the dominant molecular functions of SIPs were catalytic activity (50%), binding (28%) and molecular function regulator (10%). The main biological processes shown for SIPs were the cellular process (26%), the metabolic process (18%) and biological regulation (18%) (Figure 6A). Dominant molecular functions of SMAPs were hydrolase activity (33%), transferase activity (21%) and catalytic activity, acting on a protein (17%). The biological processes dominant in SMAPs were the cellular process (29%), biological regulation (14%) and the metabolic process (13%) (Figure 6B).

**Figure 7:**
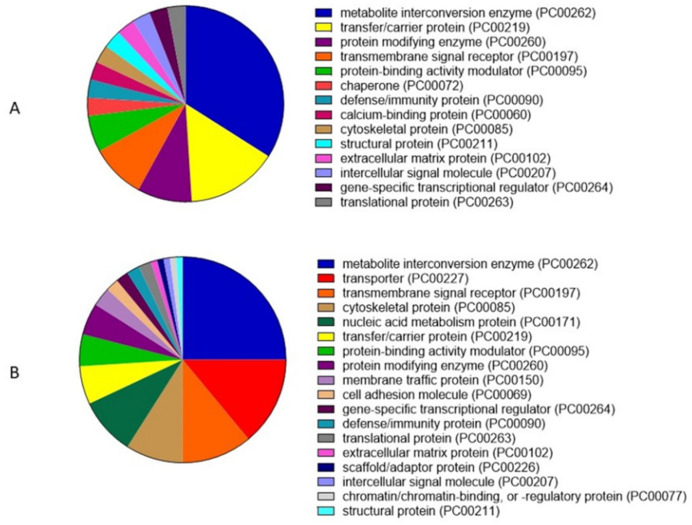
Protein classes in (**A**) sperm intracellular proteins (SIPs) and (**B**) sperm membrane-associated proteins (SMAPs) of the dog (*Canis lupus familiaris*) cauda epididymal spermatozoa. The protein class was analyzed by the PANTHER Classification System (v. 16). Each color represents percentage of protein participation in each protein class.

The protein classes recognized in SIPs after analysis of GO are mainly metabolite interconversion enzyme (34%), transfer/carrier protein (15%) and protein modifying enzyme (9%) (Figure 7A). For SMAPs, a protein class analysis showed mainly metabolite interconversion enzyme (25%), transporter (14%) and transmembrane signal receptor (11%) (Figure 7B).

**Figure 8:**
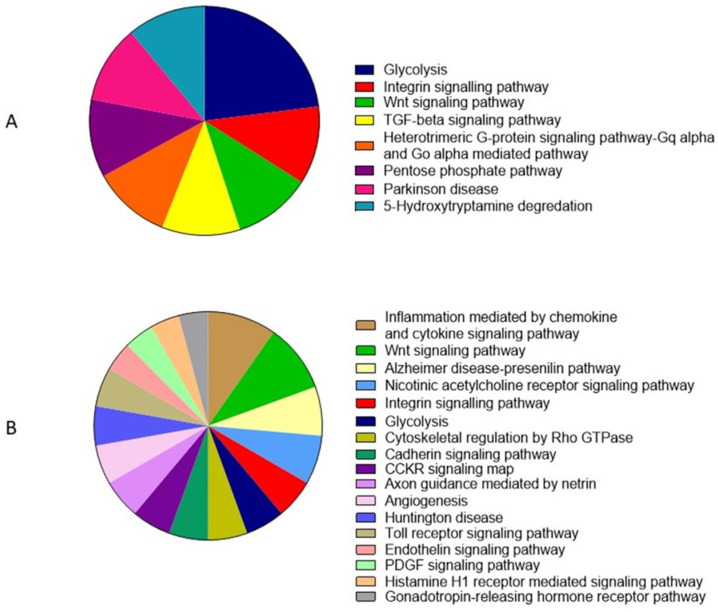
Pathways according to PANTHER Classification System (v. 16) for (**A**) sperm intracellular proteins (SIPs) and (**B**) sperm membrane-associated proteins (SMAPs) of dog (*Canis lupus familiaris*) cauda epididymal spermatozoa. Each color represents percentage of protein participation in each protein class.

The GO pathways showed mainly glycolysis (23%), the integrin signaling pathway (11%) and the Wnt signaling pathway (11%) for SIPs (Figure 8A). For the SMAPs, the main pathways were inflammation mediated by chemokine and cytokine signaling pathways (7%), the Wnt signaling pathway (7%) and the Alzheimer disease–presenilin pathway (5%) (Figure 8B).

## 4. Discussion

This is the first study presenting the isolation of intracellular and membrane-associated proteins from canine epididymal sperm and showing the comparative analysis of proteomes of the abovementioned fractions. Moreover, the present study showed the effect of sonication time and power on the percentage of damage in dog cauda epididymal spermatozoa and the sperm protein isolation.

Based on the sonication results, it is difficult to compare the results obtained in this study with the results of other scientists since different types of sonicators were used, as well as sonication parameters such as maximum power or time, which depends on the type and manufacturer of the equipment. However, it can be stated that sonication with 150 W, 10 min, can be used to isolate the head and tail of dog epididymal spermatozoa. The results are consistent with studies by other authors in which sonication was used to isolate the outer acrosomal membrane of goat ejaculated spermatozoa [11]. The acrosomal membranes were separated without damaging their structure, which was confirmed by electron microscopy. It has been shown that acrosomal membranes isolated in this way may provide good material for an analysis of the activity of specific enzymes, such as Ca^2+^ ATPase [11]. Some authors have also used sonication to separate the heads of human sperm from the tails [12]. Similar effects were achieved by Yamamoto et al. [10], who used sonication on ejaculated rat spermatozoa to remove sperm tails. In this study, a similar effect of sonication on dog epididymal spermatozoa was demonstrated, in which treatment with appropriate timing and power of sonication resulted in the effective separation of sperm heads from sperm tails. Given that sonication does not lead to damage of the sperm chromosomes, the separated sperm heads may be further used as material for in vitro fertilization [9].

It was shown that the cytoplasm from the sperm cells broken by ultrasound may leak out from the sperm cell [8]. In the current study, separate analysis was shown of the sperm intracellular proteins (SIPs) and the sperm membrane-associated proteins (SMAPs) obtained by protein extraction from the epididymal sperm residue. To date, an SDS-PAGE analysis of the SIPs and SMAPs of dog epididymal spermatozoa has not been shown. According to the authors’ knowledge, this is also the first study concerning a mass spectrometry analysis of the protein present in dog epididymal spermatozoa. Until now, mass spectrometry has allowed the identification of epididymal spermatozoa proteins of animal species, among others, for stallions [13], bulls [32], boars [33] and mice [34]. In this study, UniProt database-supported identification resulted in 42 proteins identified in the SIPs and 153 proteins in the SMAPs. Thirteen proteins were present in both fractions: *ALB* (albumin), *GPI* (Glucose-6-phosphatase isomerase), *UBA52* (Ubiqiutin-60S ribosomal protein L40), *CRISP2* (Cysteine-rich secretory protein 2), *PTGDS* (Prostaglandin-H2 D-isomerase), *LTF* (Lactotransferrin), *GPX5* (Epididymal secretory glutathione peroxidase), *ACRBP* (Acrosin-binding protein), *LCNL1* (Lipocln cytosolic FA-bd domain-containing protein), *CES5A* (Carboxylesterase 5A), *OLFM4* (Olfactomedin 4), *NPC2* (Epididymal secretory protein E1) and *HEXB* (Beta-N-acetylhexosaminidase). It is worth noting that the sperm membrane-associated fraction was much more abundant in different proteins than sperm intracellular fraction. According to proteins present in both SIPs and SMAPs, it is impossible to state if they were just contamination formed during the sperm protein isolation procedure (unavoidable) or if these proteins are really components of abovementioned sperm compartments.

The current study showed the presence of DAPs on SDS PAGE gels in both the SIPs and the SMAPs of dog epididymal spermatozoa (Figure 4, Table 2) and analyzed the potential function of these proteins in reproduction. Some of them were found in spermatozoa structures of different animal species: *LTF*, *ALB*, *OLFM4*, *CSNK1G*, *LRRN3*, *CRISP2*, *ACTB*, *UBA52*, *HEXB*, *ACRBP*, *GPX5*, *PTGDS* and *NPC2*. Three of the DAPs, however, were found only in different animal species’ reproductive tissues, such as a testis or epididymis. These included *CES5A*, WAPdcp and *LCNL1*. However, it is known that the proteins present in the epididymis may exert an impact on sperm functions [35].

The functions of DAPs, which were found among others in sperm structures of different animal species and humans, are discussed below.

Lactotransferrin (*LTF*) was found in both SIPs and SMAPs of canine epididymal sperm. A high content of *LTF* was found in the canine seminal plasma [23], and its function in reproduction is well known [27]. As it was demonstrated, *LTF* is present in high concentrations and binds to sperm in the epididymis of boars and stallions and was immunolocalized in rodent epididymis [36]. According to its binding ability to the sperm membrane [36], it was found in high amounts in our study, and it may be postulated that *LTF* also coats canine epididymal sperm plasma membrane during the sperm maturation in epididymis and might exert protective function.

In the seminal plasma of different animal species, ALBs are quite abundant proteins, and they play an important role in reproductive processes [22]. The ALBs can absorb lipid peroxides, a feature that contributes to their protective effect on membrane stability and sperm motility [37]. Albumins were found in mice sperm acrosome. They are synthesized in the epididymis and aggregate in a high molecular mass glycoprotein complex involved in sperm–egg fertilization [38].

Olfactomedin 4 (*OLFM4*) is an olfactomedin domain-containing glycoprotein [39]. OLFM-1, -2, -3, -4 are known to regulate cellular growth, differentiation and pathological processes [40]. The absence of *OLFM4* gene expression is associated with the progression of human prostate cancer, but its role and the molecular mechanisms involved in this process have not been completely understood [41]. *OLFM4* was found in human spermatozoa [42].

Peptidyl-prolyl cis-trans isomerases (*CSN*) catalyze the isomerization between the cis and trans forms of peptide bonds, which are associated with new polypeptide conformation [43,44]. In bulls, the *CSN* may exert a negative impact on spermatogenesis and sperm maturation [45]. It was found in human and rat spermatozoa [46,47].

Leucine-rich repeat protein family (*LRTP*) have been found in the testis of humans and mice [48]. A downregulation of the *LRRN3* gene in sperm of high-fertility boars was found [49], with *LRRN3* being involved in Ras-MAPK signaling [50].

*CRISP2* is a member of the cysteine-rich secretory protein (*CRISP*) family. Its expression is high in testis, and it is localized in sperm acrosome, sperm tail and the junction between germ and Sertoli cells within the seminiferous epithelium [51,52,53,54]. *CRISP2* is implicated in cell-cell adhesion and is capable of steroid binding [55,56]. It can also specifically regulate calcium flow through ryanodine receptors [57,58]. It is known that a decrease in *CRISP2* content in sperm is associated with male infertility in humans [59,60] and horses [61].

*ACTB* is the major cytoskeleton protein which is responsible for cell volume regulation [62]. The localization of the *ACT* in the flagellar and acrosomal membrane of spermatozoa suggests its role in sperm motility and capacitation [63,64,65].

*UBA52* is a low molecular weight peptide that tags other proteins for proteasomal degradation and is also involved in the regulation of other protein functions. Its role in the elimination of defective spermatozoa during transit through the epididymis has been described in humans and cattle [66,67]. Vernocchi et al. [68] indicated the presence of ubiquitinated proteins in feline epididymal sperm.

*HEXB* is a lysosomal enzyme that hydrolyses acetylglucosamine and acetylgalactosamine residues in glycoconjugates [69]. The b-hexosaminidase enzymatic ability to remove N-acetylglucosamine residues from ZP glycoproteins maintains spermatozoa properties to penetrate human oocytes [70]. It is interesting that *HEXB* has the ability to bind zinc-ions, which may influence sperm motility [71].

*ACRBP* is an acrosomal protein (also known as Sp32), and it is a binding protein specific for the precursor (proACR) and intermediate forms of *ACR* [72,73]. *ACRBP* is normally expressed exclusively in the human testis but is also expressed in a wide range of different tumor types [74,75]. *ACRBP* influences acrosin activity and acrosome formation, which is connected with sperm fertilizing ability [76].

The glutathione peroxidase (*GPX5*) is highly expressed in the epididymis of mammals, where it is secreted into the lumen, and its role is to protect sperm from lipid peroxidation. The glutathione peroxidase was found, among others, in bull, boar and dog seminal plasma [77,78,79]. Functional network analysis showed the participation of this protein in important metabolic pathways in feline SP for *GPX 5* and 6 isoforms [27]. *GPX5* was found in boar epididymal sperm [79].

*PTGDS* was found in the epididymal fluid and seminal plasma of rats [80] as well as in the seminal plasma of dogs [27]. Prostaglandin (H2) D-isomerase, expressed in different reproductive organs, binds to small nonsubstrate lipophilic molecules and may act as a scavenger for harmful hydrophobic molecules as it is potentially involved in vesicle-mediated transport and defense response [81]. Araujo et al. [24,25] described this protein as a purebred dog sperm component.

NPC intercellular cholesterol transporter 2, also called epididymal secretory protein E1, was found in SIPs and SMAPs. In general, epididymal secretory protein E1 (*CE1*/*NPC2*) is expressed based on canine genes similar to those known in humans [5]. The *CE1* protein is a highly abundant, conserved secretory protein [82]. The mRNA was found in large amounts in the epididymal duct epithelium, while the protein was found in the duct lumen [83]. Recently, *CE1* has been identified as the second gene of Niemann-Pick type C disease (*NPC2*) involved in cholesterol efflux from lysosomes [84]. It is interesting that NPC2-based proteins were found in both the SIPs and SMAPs. This may suggest its epididymal sperm membrane coating ability. Similar results were shown for the human ejaculated spermatozoa in which an *HE2* human homologue of *CE1* was present on the acrosome and equatorial region of the cells [85]. Araujo et al. [24,25] found *NPC2* as a purebred sperm component.

It may be concluded that proteins which showed high fraction-dependent (SIPs or SMAPs) content play a very important role in canine epididymal sperm metabolism, mostly by sperm protection.

This paper also discusses DAPs, which were not isolated earlier and established as sperm components. In the current study, they were shown for the first time as such. These proteins were mostly described as being produced in the epididymis.

An interesting protein with sperm quality diagnostic potential is carboxylesterase (*CES5A*). It is known to be produced in the epididymis and is required for sperm capacitation and male fertility in the rat [86]. Gene expression of *CES* was found in the rat and rabbit epididymis [87] and rat testis [88]. It is hypothesized that *CES* plays a role in the maturation of the sperm lipid membrane. The function of *CES5A* in sperm capacitation is not fully understood as it does not seem to have direct interaction with spermatozoa in the epididymal lumen but is instead thought to alter the lipid content of the luminal fluid and then, indirectly, the lipid content of the sperm membrane [86,87].

Lipocalins (*LCNL1*) typically transport or store small molecules, including vitamins, steroid hormones, odorants and various secondary metabolites [89]. Gene expression of *LCN2* was found in the mouse, ram and human epididymis [90,91,92]. Since *LCN2* could facilitate cell proliferation of castration-resistant prostate cancer via androgen receptor, *LCN2* could be a novel target in cancer therapy [93].

WAP (whey acidic protein) domain-containing protein possesses serine-type endopeptidase inhibitor activity and plays a role in biological processes such as antibacterial humoral response and innate immune response in the dog [94]. mRNA expression for this protein was found in most regions of the canine epididymis [83]. However, its function in reproduction and in canine spermatozoa was not established.

The proteins recognized as DAPs in this study and mostly described as epididymis-produced probably might be coated on sperm plasma membrane or even inserted into membrane structure during canine epididymal sperm maturation.

Some the DAPs recognized in our study such as *ALB*, *PTGDS*, *LTF* and *NPC2* were identified by mass spectrometry as components of purebred dog spermatozoa by Araujo et al. [24,25]. There have been found 47 proteins in spermatozoa, 109 in seminal plasma and 6 in both samples. Serum albumin, tubulins and acrosin binding protein were statistically relevant. Gene ontology analysis has confirmed their role in numerous important cellular biological processes.

According to the GO analysis, the proteins present in the SIPs were mainly involved in glycolysis, the integrin signaling pathway and the Wnt signaling pathway. An obligatory role for glycolysis in spermatozoa is to support its motility and capacitation process [95]. Integrin signaling pathway is involved in a broad range of processes engaged in sperm activation and fertilization [96]. For SMAPs, the main metabolic pathways were inflammation mediated by the chemokine and cytokine signaling pathway, the Wnt signaling pathway and the Alzheimer disease–presenilin pathway. The chemokine and cytokine signaling pathways contribute to testicular function and the maintenance of male reproductive health [97]. Presenilins stabilize β-catenin in the Wnt signaling pathway and regulate calcium homeostasis [98]. It was found that the presence of amyloid precursor protein (APP) is an important element of the Alzheimer disease–presenilin pathway in human sperm and testis [99,100]. The Wnt signaling pathway (showed as important for both SIPs and SMAPs) is an ancient and evolutionarily conserved pathway that regulates crucial aspects of cell fate determination, cell migration, cell polarity, neural patterning and organogenesis during embryonic development [101]. The Wnt signaling pathway has been successfully linked with the regulation of sperm maturation in the epididymis [102]. Post-transcriptional Wnt signaling influences sperm, maintaining protein homeostasis, initiating sperm motility and establishing a membrane diffusion barrier in the sperm tail [103].

As it was shown above, the protein present both in the canine epididymal sperm intracellular and membrane-associated fraction participates in important metabolic pathways regulating sperm functions and fertility. Taking into consideration the above results, some proteins may potentially be used as sperm quality markers and disease diagnostic markers of the male reproductive system.

## 5. Conclusions

The results of this study show that sonication for 10 min at 150 W can be considered for the separation of dog epididymal sperm structures and for improved protein extraction. Mass spectrometry identification and bioinformatic analysis of SIPs and SMAPs have been shown for the first time in the present study. The results show the presence of DAPs in both the sperm protein fractions, which are crucial for the functions of spermatozoa and their fertilizing ability. Finally, it has been confirmed that these proteins are implicated in important sperm metabolic pathways.

## Figures and Tables

**Table 1:** Average optical density (OD) values (mean ± SE) of differentially abundant proteins (DAPs) of the sperm intracellular proteins (SIPs) and the sperm membrane-associated proteins (SMAPs) of dog (*Canis lupus familiaris*) cauda epididymal spermatozoa (n = 15). Different superscripts within rows indicate statistically significant differences (*p* ≤ 0.001) between fractions. DAPs were marked with letters from a to j. MW—average molecular weight.

DAPs	MW (kDa)	Optical Density	
		SIPs	SMAPs
a	71.8	0.36 ± 0.012 ^a^	0.21 ± 0.006 ^b^
b	65.0	0.32 ± 0.013 ^a^	0.19 ± 0.005 ^b^
c	20.5	0.29 ± 0.008 ^a^	0.17 ± 0.007 ^b^
d	12.8	0.28 ± 0.006 ^a^	0.17 ± 0.005 ^b^
e	11.9	0.30 ± 0.007 ^a^	0.18 ± 0.005 ^b^
f	57.5	0.22 ± 0.009 ^a^	0.28 ± 0.006 ^b^
g	50.0	0.18 ± 0.006 ^a^	0.32 ± 0.008 ^b^
h	38.6	0.17 ± 0.006 ^a^	0.29 ± 0.014 ^b^
i	18.0	0.18 ± 0.007 ^a^	0.29 ± 0.006 ^b^
j	10.9	0.20 ± 0.004 ^a^	0.31 ± 0.013 ^b^

**Table 2:** Differentially abundant proteins (DAPs) of the SMAPs and SIPs present in dog (*Canis lupus familiaris*) cauda epididymal spermatozoa (n = 15). Proteins separated in SDS-PAGE were analyzed with mass spectrometry (NanoUPLC-Q-TOF/MS) and identified by UniProt identifiers. The protein score represents the quality of identification.

DAPs	Protein Name	Swiss-Prot Accession Number	Gene Symbol	Sequence Coverage (%)	Molecular Weight (kDa)	Peptide Counts (Unique)	Peptide Counts (All)	Protein Score
	SIPs							
a	Lactotransferrin	F1PR54	*LTF*	34.6	77.3	21	72	440
	Carboxylesterase 5A	Q6AW47	*CES5A*	15.1	63.6	6	44	226
b	Albumin	P49822	*ALB*	9.5	68.6	4	25	337
	Olfactomedin 4	F1PB68	*OLFM4*	4.6	54.4	1	6	236
c	Prostaglandin-H2 D-isomerase	Q9XS65	*PTGDS*	28.3	21.1	5	25	212
	Glutathione peroxidase	F1PJ71	*GPX5*	16.7	25.3	3	20	282
d	Lipocalin_cytosolic_FA-bd domain-containing protein	E2R6E0	*LCNL1*	3.7	32.6	1	7	229
	Peptidyl-prolyl cis-trans isomerase	F1PLV2	*CSNK1G1*	5.4	26.6	1	1	147
e	NPC intracellular cholesterol transporter 2	Q28895	*NPC2*	12.1	16.1	1	33	247
	Leucine rich repeat neuronal protein 3	F1PYL2	*LRRN3*	1.6	79.6	1	2	135
	SMAPs							
f	Epididymal secretory protein E1	F1PAR9	*NPC2*	33.3	20.2	4	29	470
	Lactotransferrin	F1PR54	*LTF*	17.4	77.3	12	22	335
g	Cysteine-rich secretory protein 2	A0A5F4CCD	*CRISP2*	13.5	35.4	2	5	302
	WAP domain-containing protein	E2RCT1	N/A * WAPdcp **	14.7	13.0	1	3	303
h	Actin, cytoplasmic 1	O18840	*ACTB*	5.3	41.7	4	41	378
	Ubiquitin-60S ribosomal protein L40	P63050	*UBA52*	7.0	14.7	1	4	262
i	Epididymal secretory protein E1	F1PAR9	*NPC2*	25.4	20.2	3	29	591
	Beta-N-acetylhexosaminidase	F1Q1M8	*HEXB*	6.7	38.1	1	4	276
j	Epididymal secretory protein E1	F1PAR9	*NPC2*	32.3	20.2	3	21	540
	Acrosin-binding protein	E2RNS8	*ACRBP*	1.9	61.3	1	3	231

* not available, ** protein name abbreviation in this publication.

## Data Availability

The data presented in the study are available upon request from the corresponding author.

## Supplementary Materials

The following supporting information can be downloaded at: https://www.mdpi.com/article/10.3390/ani12060772/s1, Table S1: Proteins present in epididymal sperm intracellular (SIPs) fraction of dog (*Canis lupus familiaris*), evaluated by SDS PAGE and mass spectrometry (NanoUPLC-Q-TOF/MS); Table S2: Proteins present in epididymal sperm membrane-associated fraction (SMAPs) of dog (*Canis lupus familiaris*), evaluated by SDS PAGE and mass spectrometry (NanoUPLC-Q-TOF/MS).
