# Supplementary material for: Proteomic Analysis of Intracellular and Membrane-Associated Fractions of Canine (Canis lupus familiaris) Epididymal Spermatozoa and Sperm Structure Separation
Source: Animals (Basel). 2022 Mar 18;12(6):772. doi: 10.3390/ani12060772 (PMC8944539; doi:10.3390/ani12060772)
Supplement: Supplementary file 1 [file animals-12-00772-s001.zip › Table 1 Supplementary.pdf]

Table S1. Proteins present in epididymal sperm intracellular (SIPs) fraction of dog (*Canis lupus familiaris*), evaluated by SDS PAGE and mass spectrometry (NanoUPLC-Q-TOF/MS). Proteins were identified by UniProt (Swiss-Prot accession number) and NCBI (NCBI accession number). DAPs—differentially abundant proteins in SIP fraction. Range 1–8 means protein fraction range on SDS PAGE gel.

Range	DAPs	Protein Name	Swiss-Prot Accession Number	Gene Symbol	Sequence Coverage (%)	Molecular Weight (kDa)	Peptide Counts (Unique)	Peptide Counts (all)	Protein Score
1		Mediator of RNA polymerase II transcription subunit 13	F1PTF4	*MED13L*	0.3	230.5	1	4	44
		Actin filament associated protein 1 like 2	A0A5F4CGB4	*AFAP1L2*	0.4	109.4	1	7	44
		FIP1-like 1 protein	A0A5F4CZG5	*FIP1L1*	1.0	65.5	1	1	46
2	a	Lactotransferrin	F1PR54	*LTF*	34.6	77.3	21	72	440
	b	Albumin	P49822	*ALB*	9.5	68.6	4	25	337
	b	Olfactomedin 4	F1PB68	*OLFM4*	4.6	54.4	1	6	236
	a	Carboxylesterase 5A	Q6AW47	*CES5A*	15.1	63.6	6	44	226
		Argonaute RISC component 1	F1PGP5	*AGO1*	1.3	115.9	1	2	133
		1,4-alpha-glucan branching enzyme	F1PX32	*GBE1*	1.6	80.1	1	1	124
		Chondroitin sulfate proteoglycan 5	F1P744	*CSPG5*	3.0	43.9	1	1	110
		Olfactory receptor	F1PIK8	*OR7C1*	4.1	34.9	1	1	101
		PX domain containing serine/threonine kinase like	E2QWJ5	*PXK*	2.1	63.4	1	1	96
		Tubulin polymerization promoting protein	J9NYH4	*TPPP*	1.8	65.3	1	1	96
		NEDD4 binding protein 2	A0A5F4CIQ4	*N4BP2*	0.6	189.1	1	1	90
		Non-specific serine/threonine protein kinase	A0A5F4D7J3	*CDC42BPA*	0.5	204.1	1	1	87
		RHEB like 1	E2QTC6	*RHEBL1*	3.7	21.0	1	6	83
		Growth differentiation factor 5	E2R0W8	*GDF5*	2.6	55.1	1	1	83
		Nuclear receptor binding SET domain protein	J9NUG7	*NSD2*	1.0	149.9	1	1	71
3		Beta-N-acetylhexosaminidase	F1Q1M8	*HEXB*	15.8	38.1	3	8	325
		2-phospho-D-glycerate hydro-lyase	F1PCH3	*ENO1*	6.3	21.2	1	1	167
		Aldehyde dehydrogenase 1 family member A1	J9P9J4	*ALDH1A1*	9.7	53.0	3	6	163
		Hyaluronidase	E2RCK6	*SPAM1*	8.3	61.7	3	5	120
		Lactotransferrin	A0A5F4BVF3	*LTF*	3.0	68.8	1	1	95
		Glucose-6-phosphate isomerase	E2R2C3	*GPI*	3.0	63.0	1	2	89

4		Acrosin	A0A5F4C1D8	*ACR*	4.7	46.0	1	1	69
		ArfGAP with RhoGAP domain, ankyrin repeat and PH domain 1	A0A5F4CQA6	*ARAP1*	0.5	154.0	1	3	67
5	c	Glutathione peroxidase	F1PJ71	*GPX5*	16.7	25.3	3	20	282
		Cysteine rich secretory protein 2	A0A5F4CCD0	*CRISP2*	10.6	35.4	2	14	250
	c	Prostaglandin-H2 D-isomerase	Q9XS65	*PTGDS*	28.3	21.1	5	25	212
		X-prolyl aminopeptidase 3	A0A5F4DFA0	*XPNPEP3*	2.1	52.6	1	1	82
6		Prostaglandin-H2 D-isomerase	Q9XS65	*PTGDS*	28.3	21.1	6	69	368
		tRNA-yW synthesizing protein 5	E2R278	*TYW5*	2.2	36.6	1	1	85
7	e	NPC intracellular cholesterol transporter 2	Q28895	*NPC2*	12.1	16.1	1	33	247
	d	Lipocln_cytosolic_FA-bd_dom domain-containing protein	E2R6E0	*LCNL1*	3.7	32.6	1	7	229
	d	Peptidyl-prolyl cis-trans isomerase	F1PLV2	*CSNK1G1*	5.4	26.6	1	1	147
	e	Leucine rich repeat neuronal 3	F1PYL2	*LRRN3*	1.6	79.6	1	2	135
8		Acrosin-binding protein	E2RNS8	*ACRBP*	1.9	61.3	1	18	375
		Glutathione peroxidase	F1PJ71	*GPX5*	5.4	25.3	1	2	171
		Piezo-type mechanosensitive ion channel component	A0A5F4BUL6	*PIEZO2*	0.3	311.4	1	2	166
		Ubiquitin-60S ribosomal protein L40	P63050	*UBA52*	12.5	14.7	1	1	124
		GTPase activating Rap/RanGAP domain like 3	A0A5F4CW91	*GARNL3*	0.9	110.6	1	2	122
		Activator of HSP90 ATPase activity 1	E2RSU7	*AHSA1*	4.7	38.3	1	1	120
		Cone-rod homeobox protein	Q8SQ03	*CRX*	6.4	32.3	1	1	115
		Taste receptor type 2	Q2ABC9	*TAS2R41*	2.6	35.5	1	1	109
		Angiomotin like 1	A0A5F4D0L1	*AMOTL1*	3.7	100.7	2	2	102
		Calbindin	F6UNU8	*CALB1*	3.5	29.9	1	2	101
		Collagen type IV alpha 4 chain	A0A5F4C7V5	*COL4A4*	1.1	159.4	1	1	91
		Glycine cleavage system P protein	A0A5F4C8B1	*GLDC*	1.1	113.3	1	1	86
		Integrator complex subunit 10	E2RI21	*INTS10*	1.3	82.3	1	2	82
		Glycerol-3-phosphate acyltransferase 2, mitochondrial	A0A5F4CIW1	*GPAT2*	2.1	89.5	1	1	79
