# Supplementary material for: Proteomic Analysis of Intracellular and Membrane-Associated Fractions of Canine (Canis lupus familiaris) Epididymal Spermatozoa and Sperm Structure Separation
Source: Animals (Basel). 2022 Mar 18;12(6):772. doi: 10.3390/ani12060772 (PMC8944539; doi:10.3390/ani12060772)
Supplement: Supplementary file 1 [file animals-12-00772-s001.zip › Table 2 Supplementary.pdf]

Table S2. Proteins present in epididymal sperm membrane-associated fraction (SMAPs) of dog (*Canis lupus familiaris*), evaluated by SDS PAGE and mass spectrometry (NanoUPLC-Q-TOF/MS). Proteins were identified by UniProt (Swiss-Prot accession number) and NCBI (NCBI accession number). DAPs—differentially abundant proteins in SMAP fraction. Range 1–8 means protein fraction range on SDS PAGE gel.

Range	DAPs	Protein Name	Swiss-Prot Accession Number	Gene Symbol	Sequence Coverage (%)	Molecular Weight (kDa)	Peptide Counts (Unique)	Peptide Counts (All)	Protein Score
1		NPC intracellular cholesterol transporter 2	Q28895	*NPC2*	15.4	16.1	1	4	229
		Arginine esterase	P09582	*N/A*	5.4	28.7	1	2	200
		Lactotransferrin	A0A5F4BVF3	*LTF*	6.7	68.8	2	5	170
		F-actin monooxygenase	A0A5F4D7I6	*MICAL1*	0.7	111.2	1	4	163
		A-kinase anchoring protein 13	F1Q074	*AKAP13*	0.3	301.7	1	1	143
		Phosphatidylinositol 4-kinase alpha	F1PMC5	*PI4KA*	1.1	236.4	1	1	96
		ATP binding cassette subfamily C member 4	A0A5F4CYQ3	*ABCC4*	1.9	146.1	1	1	85
		Transient receptor potential cation channel subfamily C member 5	F1PA53	*TRPC5*	2.3	111.4	1	1	81
		GTP binding protein 2	E2RC60	*GTPBP2*	1.3	65.7	1	1	79
2		Epididymal secretory protein E1	F1PAR9	*NPC2*	32.3	20.2	3	27	564
		NPC intracellular cholesterol transporter 2	Q28895	*NPC2*	21.5	16.1	2	23	552
		Alpha fetoprotein	F2Z4Q6	*AFP*	38.6	72.0	18	72	462
		Lactotransferrin	F1PR54	*LTF*	15.4	77.3	9	25	453
		Lipocln_cytosolic_FA-bd_dom domain-containing protein	E2R6E0	*LCNL1*	3.4	32.6	1	5	405
		Boule homolog, RNA binding protein	A0A5F4C9S3	*BOLL*	1.9	39.7	1	4	179
		Olfactory receptor	F1PCR4	*OR2AE1*	3.8	36.1	1	1	161
		Cysteine rich secretory protein 2	A0A5F4D6X1	*CRISP2*	8.4	36.1	1	2	143
		Terminal uridylyl transferase 7	A0A5F4D1U6	*TUT7*	1.3	169.8	1	1	135
		Glutamine--fructose-6-phosphate transaminase (isomerizing)	J9P432	*GFPT1*	1.2	76.1	1	1	134
		Importin subunit alpha	F1PV58	*KPNA3*	4.6	55.1	1	1	125
		Olfactory receptor-like protein DTMT	P30955	*N/A*	13.4	35.2	1	2	124
		Ig-like domain-containing protein	J9NSQ1	*N/A*	15.8	12.9	1	2	119

		Inositol 1,4,5-trisphosphate receptor type 2	A0A5F4CFY8	*ITPR2*	0.9	278.8	1	1	117
		Peptidylglycine alpha-amidating monooxygenase	F6X870	*PAM*	3.0	91.6	1	2	108
		GLI family zinc finger 1	A0A5F4D967	*GLI1*	0.7	121.0	1	5	107
		Olfactory receptor family 6 subfamily C member 3I	A0A5F4CFE1	*OR6C3I*	8.0	35.5	1	1	100
		Potassium voltage-gated channel subfamily Q member 4	F1Q3A1	*KCNQ4*	3.0	79.2	1	1	96
		Septin-type G domain-containing protein	A0A5F4C9N8	*SEPTIN9*	1.4	65.5	1	3	91
		Toll like receptor 4	F1PDB9	*TLR4*	1.0	96.0	1	9	89
		Astrotactin 1	A0A5F4D0C2	*ASTN1*	2.1	140.9	1	2	86
		Codanin 1	F1PXQ3	*CDAN1*	1.9	131.3	1	2	84
		Malignant T-cell-amplified sequence	J9P226	*LOC487150*	11.0	20.6	1	1	84
		GIT ArfGAP 1	A0A5F4DJN3	*GIT1*	3.3	84.4	1	1	80
		Netrin receptor UNC5	E2RC29	*UNC5B*	4.9	103.1	2	2	75
		Olfactory receptor	F1PE91	*OR6C69G*	8.4	34.9	1	1	74
3	f	Epididymal secretory protein E1	F1PAR9	*NPC2*	33.3	20.2	4	29	470
	f	Lactotransferrin	F1PR54	*LTF*	17.4	77.3	12	22	335
	g	WAP domain-containing protein	E2RCT1	*N/A*	14.7	13.0	1	3	303
	g	Cysteine rich secretory protein 2	A0A5F4CCD0	*CRISP2*	13.5	35.4	2	5	302
		A-kinase anchoring protein 13	F1Q074	*AKAP13*	0.3	301.7	1	2	183
		Pyruvate kinase	A0A5F4CGU2	*PKLR*	6.8	60.2	1	3	175
		Phospholipase A2 receptor 1	A0A5F4D8I6	*PLA2R1*	0.5	160.5	1	3	162
		Nik related kinase	F1PB88	*NRK*	0.5	171.5	1	1	136
		Transmembrane protein 115	E2QWQ9	*TMEM115*	11.1	38.2	1	1	133
		Alpha-1,3-mannosyl-glycoprotein 4-beta-N-acetylglucosaminyltransferase B	A0A5F4CBI5	*MGAT4B*	4.3	63.9	1	1	127
		Phosphatidylinositol glycan anchor biosynthesis class S	F6X735	*PIGS*	5.2	61.5	1	2	122
		LDL receptor related protein 2	F1PIB4	*LRP2*	0.6	520.6	2	2	120

		Codanin 1	F1PXQ3	*CDAN1*	1.9	131.3	1	3	118
		GRB2 associated regulator of MAPK1 subtype 1	A0A5F4DGU3	*GAREM1*	1.1	87.4	1	1	106
		Docking protein 1	A0A5F4BR19	*DOK1*	21.1	19.2	1	1	104
		Protein inhibitor of activated STAT 3	F1Q1E9	*PIAS3*	6.8	64.6	1	1	100
		Phospholipid-transporting ATPase	J9P2W7	*ATP8A1*	1.9	132.9	1	2	94
		ElaC homolog protein 2	A0A5F4CLL9	*ELAC2*	1.1	91.3	1	1	94
		EFR3 homolog B	F1PMW4	*EFR3B*	0.9	103.0	1	1	94
		Family with sequence similarity 181 member B	J9NZN3	*FAM181B*	8.4	40.8	2	2	92
		Diacylglycerol kinase	A0A5F4C8J4	*DGKH*	1.9	134.6	1	1	84
		Zona pellucida sperm-binding protein 3	P48831	*ZP3*	2.1	47.4	1	1	79
		VPS37 C-terminal domain-containing protein	E2QZC7	*VPS37B*	10.1	31.4	1	1	78
		Olfactory receptor	E2R1U1	*OR9G10*	14.5	34.8	1	2	78
		CCCTC-binding factor like	E2RLE7	*CTCFL*	2.0	35.4	1	27	75
		YEATS domain containing 4	E2QSI2	*YEATS4*	2.6	26.5	1	1	71
4		Lactotransferrin	A0A5F4BVF3	*LTF*	11.3	68.8	6	26	382
	h	Actin, cytoplasmic 1	O18840	*ACTB*	5.3	41.7	4	41	378
		NPC intracellular cholesterol transporter 2	Q28895	*NPC2*	30.2	16.1	3	9	301
		von Willebrand factor A domain containing 5A	A0A5F4CPY7	*VWA5A*	0.8	89.2	1	4	277
	h	Ubiquitin-60S ribosomal protein L40	P63050	*UBA52*	7.0	14.7	1	4	262
		Albumin	P49822	*ALB*	8.9	68.6	5	14	258
		Epididymal secretory protein E1	F1PAR9	*NPC2*	27.5	20.2	3	8	255
		Cysteine rich secretory protein 2	A0A5F4CCD0	*CRISP2*	8.4	35.4	2	2	238
		Lactotransferrin	F1PR54	*LTF*	14.4	77.3	9	21	229
		Prostaglandin-H2 D-isomerase	Q9XS65	*PTGDS*	7.3	21.1	1	2	221
		WAP domain-containing protein	E2RCT1	*N/A*	9.5	13.0	2	5	206
		A-kinase anchoring protein 13	F1Q074	*AKAP13*	0.3	301.7	1	4	194
		Pleckstrin homology domain containing A4	A0A5F4BQH3	*PLEKHA4*	0.9	85.1	1	1	180
		Senataxin	A0A5F4CUE8	*SETX*	0.4	300.1	1	3	176
		Solute carrier family 4 member 9	J9P3D0	*SLC4A9*	1.1	98.1	1	3	174

	1-phosphatidylinositol 4,5-bisphosphate phosphodiesterase gamma	F1PS03	*PLCG1*	1.2	140.4	1	1	171
	Cyclic nucleotide binding domain containing 1	F1Q3I2	*CNBD1*	1.0	58.5	1	1	165
	Pyruvate dehydrogenase E1 component subunit beta	J9P208	*N/A*	3.0	36.3	1	1	153
	Acrosin	A0A5F4C1D8	*ACR*	2.1	46.0	1	1	152
	Epididymal secretory glutathione peroxidase	O46607	*GPX5*	2.7	25.3	1	2	153
	Metaxin	F1PSR1	*MTX3*	9.0	32.2	1	1	147
	Olfactory receptor	E2R8V1	*OR4G18*	1.9	35.5	1	1	144
	RNA polymerase II subunit C	E2RQ42	*POLR2C*	2.5	32.1	1	1	142
	Reverse transcriptase domain-containing protein	A0A5F4DC84	*N/A*	1.1	78.7	1	1	130
	CE10 protein	Q9XSV4	*ce10*	9.1	12.8	1	5	129
	Proline-serine-threonine phosphatase interacting protein 1	A0A5F4C854	*PSTPIP1*	3.6	15.9	1	1	123
	Component of oligomeric Golgi complex 7	A0A5F4D723	*COG7*	1.4	83.2	1	1	122
	Cullin 7	J9NWA1	*CUL7*	0.6	176.9	1	1	114
	TEPSIN adaptor related protein complex 4 accessory protein	A0A5F4D252	*TEPSIN*	1.3	61.4	1	1	106
	Ezrin	E2RSI6	*EZR*	1.5	69.4	1	1	92
	Integrin subunit alpha V	A0A5F4C9R4	*ITGAV*	1.5	114.4	1	1	91
	FYVE, RhoGEF and PH domain containing 6	A0A5F4CU74	*FGD6*	1.4	148.3	1	3	86
	Tubulin tyrosine ligase like 5	A0A5F4CSA7	*TTLL5*	0.9	142.9	1	1	79
	Tudor domain containing 9	J9P8U1	*TDRD9*	1.3	151.1	1	2	76
	TAO kinase 3	A0A5F4CM71	*TAOK3*	1.4	95.4	1	1	71
	Transmembrane protein 117	J9P2K4	*TMEM117*	2.6	59.6	1	1	71
	Solute carrier family 15 member 1	Q8WMX5	*SLC15A1*	0.3	78.7	1	2	70
5	Cysteine rich secretory protein 2	A0A5F4D6X1	*CRISP2*	8.4	36.1	1	5	361
	Lactotransferrin	F1PR54	*LTF*	5.4	77.3	3	10	252

	Lipocln_cytosolic_FA-bd_dom domain-containing protein	E2R6E0	*LCNL1*	3.7	32.6	1	1	217
	Albumin	P49822	*ALB*	12.5	68.6	5	6	205
	Acrosin	A0A5F4C1D8	*ACR*	5.9	46.0	1	1	198
	ATP synthase subunit beta	A0A5F4DJV1	*ATP5F1B*	2.4	68.0	1	1	196
	Glucose-6-phosphate isomerase	E2R2C3	*GPI*	3.2	63.0	1	6	184
	Epididymal secretory glutathione peroxidase	O46607	*GPX5*	15.8	25.3	2	3	179
	Carboxylesterase 5A	Q6AW47	*CES5A*	3.7	63.6	1	3	168
	A-kinase anchoring protein 13	F1Q074	*AKAP13*	0.3	301.7	1	2	151
	Actin, cytoplasmic 1	O18840	*ACTB*	2.4	41.7	1	1	146
	Fructose-bisphosphate aldolase	J9P7A6	*ALDOA*	5.7	38.2	1	1	139
	Interleukin 4 induced 1	A0A5F4DIG1	*IL4I1*	8.1	63.7	3	3	125
	C1q and TNF related 5	J9NSY4	*C1QTNF5*	16.7	12.2	1	1	121
	ANK_REP_REGION domain-containing protein	H9GWX7	*LOC608294*	4.9	37.1	1	2	119
	T-cell surface glycoprotein CD8 alpha chain	P33706	*CD8A*	5.0	26.0	1	2	110
	Tumor susceptibility 101	A0A5F4DKE2	*TSG101*	9.8	40.1	1	1	94
	Atlastin GTPase 1	A0A5F4CRL0	*ATL1*	5.8	63.1	1	1	92
	Ribonuclease	E2RQK5	*RNASEH2A*	5.2	53.5	1	2	92
	Stathmin domain containing 1	A0A5F4DC42	*STMND1*	5.5	40.4	1	1	92
	Tubulin alpha chain	F1PE21	*LOC477570*	3.1	50.0	1	1	89
	Ig-like domain-containing protein	E2RHT2	*CD8B*	1.9	23.6	1	2	88
	NPC intracellular cholesterol transporter 2	Q28895	*NPC2*	15.4	16.1	1	1	79
	Phosphate transporter	A0A5F4DI30	*SLC20A2*	6.4	66.0	1	1	75
	Titin	A0A5F4BU36	*TTN*	0.1	3011.0	5	10	74
	Neutral ceramidase	A0A5F4D9P9	*ASAH2*	3.5	84.6	1	1	72
6	Lactotransferrin	F1PR54	*LTF*	20.5	77.3	13	56	516
	NPC intracellular cholesterol transporter 2	Q28895	*NPC2*	15.4	16.1	1	10	496

Lipocln_cytosolic_FA-bd_dom domain-containing protein	E2R6E0	*LCNL1*	3.7	32.6	1	10	380
Prostaglandin-H2 D-isomerase	Q9XS65	*PTGDS*	17.8	21.1	3	8	340
Albumin	P49822	*ALB*	7.6	68.6	3	10	320
Ig-like domain-containing protein	J9NSQ1	*N/A*	15.8	12.9	1	3	282
Beta-N-acetylhexosaminidase	F1Q1M8	*HEXB*	6.7	38.1	1	2	247
Sulfhydryl oxidase	F1PLT8	*QSOX1*	2.6	63.1	1	1	235
Olfactomedin 4	F1PB68	*OLFM4*	4.2	54.4	2	5	230
A-kinase anchoring protein 13	F1Q074	*AKAP13*	0.3	301.7	1	6	229
Alkaline phosphatase	A0A5F4DGF5	*ALPL*	1.8	62.9	1	1	224
NTR domain-containing protein	J9NZH4	*LOC102154527*	8.6	25.3	1	1	184
GLI family zinc finger 1	A0A5F4D967	*GLI1*	0.7	121.0	1	22	182
Olfactory receptor	F1PCR4	*OR2AE1*	3.8	36.1	1	3	172
Cysteine rich secretory protein 2	A0A5F4CCD0	*CRISP2*	13.5	35.4	2	5	158
von Willebrand factor A domain containing 3A	F1PFJ3	*VWA3A*	1.2	134.3	1	1	154
Boule homolog, RNA binding protein	A0A5F4C9S3	*BOLL*	1.9	39.7	1	7	151
Membrane associated guanylate kinase, WW and PDZ domain containing 2	J9NWS2	*MAGI2*	0.6	129.2	1	7	151
Leucine rich repeat containing 58	E2R023	*LRRC58*	1.4	61.0	1	1	139
Acyltransferase	F1PRI9	*DGAT2*	1.9	47.8	1	15	113
Myotubularin related protein 11	E2R1T1	*MTMR11*	2.1	78.3	1	1	110
Rhomboid-related protein 2	E2QY16	*RHBDL2*	5.6	34.3	1	1	102
Epididymal secretory glutathione peroxidase	O46607	*GPX5*	6.8	25.3	1	1	101
Claudin domain containing 1	F1PYH4	*CLDND1*	6.3	39.0	1	1	100
Phosphatidylinositol-4,5-bisphosphate 4-phosphatase	F1PQN6	*OSGEP*	6.2	36.5	1	1	98
Protein kinase domain-containing protein	A0A5F4D0I1	*CSNK2A1*	5.9	40.7	1	2	93
Taste receptor type 2	A0A5F4BRQ1	*TAS2R4*	2.8	33.0	1	4	91
FA complementation group M	J9NY43	*FANCM*	0.9	229.4	1	1	87
Hyperpolarization activated cyclic nucleotide gated potassium channel 4	J9PAZ6	*HCN4*	4.9	61.2	1	1	86

		U2 snRNP associated SURP domain containing	E2R1V3	*U2SURP*	1.4	118.3	1	2	80
		Ectonucleoside triphosphate diphosphohydrolase 7	F1PKY7	*ENTPD7*	3.0	69.0	1	1	78
		Semaphorin 6B	F1PX52	*SEMA6B*	2.7	80.9	1	1	78
		Adenylate cyclase type 1	J9NY35	*ADCY1*	2.3	126.1	1	2	76
		Olfactory receptor	A0A5F4DHC7	*OR13D1B*	12.7	35.5	1	1	72
		Dispatched RND transporter family member 3	A0A5F4CKV9	*DISP3*	1.8	144.3	1	3	70
7	i	Epididymal secretory protein E1	F1PAR9	*NPC2*	25.4	20.2	3	29	591
		Albumin	P49822	*ALB*	14.5	68.6	8	35	550
		Lactotransferrin	F1PR54	*LTF*	21.5	77.3	14	56	543
		NPC intracellular cholesterol transporter 2	Q28895	*NPC2*	21.5	16.1	2	9	541
		Lipocln_cytosolic_FA-bd_dom domain-containing protein	E2R6E0	*LCNL1*	3.7	32.6	1	8	424
		Sulfhydryl oxidase	F1PLT8	*QSOX1*	2.6	63.1	1	3	364
	i	Beta-N-acetylhexosaminidase	F1Q1M8	*HEXB*	6.7	38.1	1	4	276
		Epididymal secretory glutathione peroxidase	O46607	*GPX5*	6.8	25.3	1	5	264
		Olfactomedin 4	F1PB68	*OLFM4*	4.2	54.4	1	2	240
		Cysteine rich secretory protein 2	A0A5F4CCD0	*CRISP2*	4.8	35.4	1	4	220
		Olfactory receptor	F1PCR4	*OR2AE1*	3.8	36.1	1	1	202
		Carboxylesterase 5A	Q6AW47	*CES5A*	3.5	63.6	2	109	194
		Senataxin	A0A5F4CUE8	*SETX*	0.3	300.1	1	1	187
		von Willebrand factor A domain containing 3A	F1PFJ3	*VWA3A*	1.2	134.3	1	2	182
		Boule homolog, RNA binding protein	A0A5F4C9S3	*BOLL*	1.9	39.7	1	3	172
		Membrane associated guanylate kinase, WW and PDZ domain containing 2	J9NWS2	*MAGI2*	0.6	129.2	1	2	172
		A-kinase anchoring protein 13	F1Q074	*AKAP13*	0.3	301.7	1	5	169
		Olfactory receptor	J9P4T0	*OR5AC26*	6.4	37.3	1	3	137
		Rho GTPase activating protein 24	A0A5F4BYE4	*ARHGAP24*	1.9	76.2	1	1	131
		Adhesion G protein-coupled receptor A2	J9NRV9	*ADGRA2*	2.0	140.9	1	1	131

		U2 snRNP associated SURP domain containing	E2R1V3	*U2SURP*	1.4	118.3	1	1	125
		Interleukin-1 receptor-associated kinase 4	E2RDL0	*IRAK4*	2.8	51.4	1	1	115
		Glutamine--fructose-6-phosphate transaminase (isomerizing)	J9P432	*GFPT1*	1.2	76.1	1	3	103
		RUN domain containing 1	F1PAD7	*RUNDC1*	1.3	67.6	1	1	97
		Codanin 1	F1PXQ3	*CDAN1*	1.9	131.3	1	4	95
		Solute carrier family 14 member 1	A0A5F4CTT5	*SLC14A1*	2.5	53.7	1	1	95
		TraB domain containing 2A	J9NY87	*TRABD2A*	3.0	57.8	1	1	92
		Phospholipase A2 receptor 1	A0A5F4D8I6	*PLA2R1*	0.5	160.5	1	1	91
		Galectin	A0A5F4C4R0	*LGALS9*	3.5	41.4	1	1	91
		Calcium dependent secretion activator 2	A0A5F4D577	*CADPS2*	1.1	147.2	1	1	90
		Dispatched RND transporter family member 3	A0A5F4CKV9	*DISP3*	1.8	144.3	1	2	89
		Ig-like domain-containing protein	A0A5F4CQU2	*DLA-DMB*	4.1	27.4	1	4	88
		Ectonucleoside triphosphate diphosphohydrolase 1	A0A5F4CCH2	*ENTPD1*	3.7	55.1	1	2	86
		zf-3CxxC domain-containing protein	J9NXR0	*RTP5*	1.4	60.5	1	1	80
		Desmoglein-1	Q9GKQ8	*DSG1*	4.0	113.9	1	2	80
		Myosin IE	A0A5F4CHW1	*MYO1E*	1.3	121.9	1	1	80
		Nik related kinase	F1PB88	*NRK*	0.5	171.5	1	2	79
		Caspase recruitment domain family member 6	F1PIZ1	*CARD6*	0.7	122.2	1	1	79
		Cation-transporting ATPase	F1PRT7	*ATP13A4*	0.7	134.0	1	3	76
		Transporter 1, ATP binding cassette subfamily B member	F1PTP4	*TAP1*	3.4	75.4	1	1	75
		Calcium-transporting ATPase	J9P2B9	*ATP2A3*	2.7	109.6	1	1	74
		Signal recognition particle subunit SRP72	P33731	*SRP72*	3.7	74.5	1	1	73
		Histone H2A	F1P790	*H2AW*	21.5	14.2	1	1	72
8	j	Epididymal secretory protein E1	F1PAR9	*NPC2*	32.3	20.2	3	21	540
		Lactotransferrin	A0A5F4BVF3	*LTF*	12.6	68.8	6	22	497
		Cysteine rich secretory protein 2	A0A5F4D6X1	*CRISP2*	8.4	36.1	1	4	330
		A-kinase anchoring protein 13	F1Q074	*AKAP13*	0.3	301.7	1	5	237

j	Acrosin-binding protein	E2RNS8	*ACRBP*	1.9	61.3	1	3	231
	NTR domain-containing protein	J9NZH4	*LOC10215 4527*	8.6	25.3	1	1	165
	Olfactory receptor family 56 subfamily B member 1	F6XR78	*OR56B1*	3.1	35.8	1	1	104
	Fibrocystin	E2RK30	*PKHD1*	2.3	447.6	6	6	103
	Adhesion G protein-coupled receptor A2	J9NRV9	*ADGRA2*	2.0	140.9	2	3	101
	CD109 molecule	A0A5F4CP99	*CD109*	1.8	169.3	1	3	100
	Dual oxidase maturation factor 1	A0A5F4BRG 3	*DUOXA1*	19.9	32.8	1	1	93
	Solute carrier family 16 member 6	F1Q090	*SLC16A6*	5.5	57.1	1	1	91
	Interferon alpha inducible protein 6	E2RSX9	*IFI6*	55.4	13.3	1	1	90
	WAP domain-containing protein	E2RCT1	*N/A (WAPdcp)*	14.7	13.0	1	2	85
	Myosin heavy chain 7	E2RJD1	*MYH7*	1.6	203.4	1	2	84
	Protein inhibitor of activated STAT 3	F1Q1E9	*PIAS3*	6.8	64.6	2	2	82
	DOP1 leucine zipper like protein B	E2RIH1	*DOP1B*	1.2	253.6	1	1	77
	Tuftelin-interacting protein 11	A1XD97	*TFIP11*	3.2	96.6	1	1	71
